# The Relationship between the p.V37I Mutation in *GJB2* and Hearing Phenotypes in Chinese Individuals

**DOI:** 10.1371/journal.pone.0129662

**Published:** 2015-06-10

**Authors:** Shasha Huang, Bangqing Huang, Guojian Wang, Yongyi Yuan, Pu Dai

**Affiliations:** 1 Department of Otolaryngology, PLA General Hospital, Do.28 Fuxing Road, Beijing, People’s Republic of China; 2 Department of Otolaryngology, Hainan Branch of PLA General Hospital, Haitang Bay, Sanya, People’s Republic of China; Pasteur Institute of Lille, FRANCE

## Abstract

The most common cause of nonsyndromic autosomal recessive hearing loss is mutations in *GJB2*. The mutation spectrum and prevalence of mutations vary significantly among ethnic groups, and the relationship between p.V37I mutation in *GJB2* and the hearing phenotype is controversial. Among the 3,864 patients in this study, 106 (2.74%) had a homozygous p.V37I variation or a compound p.V37I plus other *GJB2* pathogenic mutation, a frequency that was significantly higher than that in the control group (600 individuals, 0%). The hearing loss phenotype ranged from mild to profound in all patients with the homozygous p.V37I variation or compound p.V37I plus other *GJB2* pathogenic mutation. There was no difference in the distribution of the hearing level in the group with the homozygous p.V37I variation and the group with the compound p.V37I variation plus pathogenic mutation. Most patients (66.04%) with the V37I-homozygous variation or p.V37I plus other pathogenic mutation had a mild or moderate hearing level. This study found a definite relationship between p.V37I and deafness, and most patients who carried the pathogenic combination with p.V37I mutation had mild or moderate hearing loss. Therefore, otolaryngologists should consider that the milder phenotype might be caused by the *GJB2* p.V37I mutation.

## Introduction

Hearing impairment is the most common neurosensory disorder in humans, with reported incidences ranging from 1 in 300 to 1 in 1,000 children. Genetics plays a role in approximately half of the cases, including syndromic and nonsyndromic forms. Nonsyndromic deafness accounts for 60–70% of inherited hearing impairment cases, with autosomal recessive being the most common type of inheritance. For many populations, the most common cause of nonsyndromic autosomal recessive hearing loss is mutation in connexin 26, a gap junction protein encoded by the *GJB2* gene [1–3]. A few mutations in *GJB2* have also been reported to cause dominant nonsyndromic or syndromic hearing loss [4,5]. Connexins are transmembrane proteins. Six monomers of connexin proteins associate to form a transmembrane hexameric gap junction hemi-channel called a connexon. Connexons embedded in the surfaces of adjacent cells associate to form an intercellular channel. In the inner ear, connexin 26 can associate with other connexins to form heteromeric connexons. Gap junction channels can be homotypic or heterotypic. Connexin 26 gap junction channels recycle potassium ions as part of a mechanism of auditory signal transduction in the inner ear.

To date, more than 150 mutations, polymorphisms, and unclassified variants have been described in the *GJB2* gene. The mutation spectrum and prevalence of mutations vary significantly among ethnic groups. Three mutations, c.235delC, c.35delG, and c. 167delT, are the most frequent mutations in Asian, Caucasian and Ashkenazi Jewish populations respectively [2,3,6]. The p.V37I variation in *GJB2* is highly prevalent in East Asian deafness, but there is a controversial relationship between some mutations, including p.V37I(c.109G>A), and the hearing phenotype. Few systematic studies have examined the role of p.V37I in the pathogenesis of hearing loss. In the present study, 3,864 patients with nonsyndromic sensorineural hearing loss and 600 normal-hearing individuals were included to investigate the nature of the p.V37I variant and provide possible genetic testing and counseling for hearing loss patients with this variant in China.

## Patients and Methods

### Patients

In total, 3,864 patients with nonsyndromic sensorineural hearing loss (from unrelated families) were reached through the Otolaryngology Department of the Chinese PLA General Hospital and were included in the current study. A complete history, physical and otoscopic examinations, and audiological testing, including pure tone audiometry, tympanometry, or auditory brainstem response, were carried out in all of the patients, and diagnosed as sensorineural hearing loss. All 3,864 patients were sequence analyzed for the exon 2 of *GJB2*. High-resolution temporal bone computed tomography (CT) was available for all of the recruited patients to rule out inner ear malformations, including enlarged vestibular aqueduct syndrome patients. Additionally, all of the patients had no skin problems or other clinical abnormalities and no m.A1555G or m.C1494T mutation in mitochondrial *12S rRNA*.

Fully informed written consent was obtained from all subjects or, in the case of children, from their guardians. The study was approved by the Chinese PLA General Hospital Research Ethics Committee. To identify the p.V37I prevalence in normal individuals, 600 individuals were included as the control group and were sequence analyzed for the exon 2 of *GJB2*. The age ranged from 2 to 60 years, only 42 individuals were aged less than 18 years, and the median age was 32 years. Audiological testing was carried out in all of the individuals, revealing normal hearing or conductive hearing loss but normal bone conduction threshold.

### Clinical evaluation

No patient was found to have any goiter symptom or sign or syndromic symptoms in other systems. The pure-tone average (PTA) was calculated as the average of the threshold measured at 0.5, 1.0, 2.0, and 4.0 kHz and was used to compare subgroups of patients. The level of hearing loss, in terms of PTA, was described as follows: normal hearing, < 20 dB; mild hearing impairment, 21–40 dB; moderate hearing impairment, 41–70 dB; severe hearing impairment, 71–90 dB; and profound hearing impairment, > 90 dB.

### Statistical testing

Chi-squared test (R × C table) was used to compare the distribution of the p.V37I mutation in the control and patient groups. The hearing level between the different groups was included in the nonparametric randomized block design information. The Friedman rank-sum test (M-test) was used to identify significant differences in hearing level among the groups. Statistical analyses were performed using the SPSS (Statistical Package for the Social Sciences) 16.0 software. A P-value of 0.05 or less was deemed to indicate statistical significance.

## Results

Among the 3,864 patients, 314 had one or two GJB2 p.V37I variations (8.13%), and the mean age of the 314 patients was 19.6 years (range: 7 months to 54 years). Heterozygous p.V37I variation, homozygous p.V37I variation, and compound p.V37I plus other *GJB2* pathogenic mutation (Table 1) were detected in 208 patients (5.38%), 57 patients (1.48%), and 49 patients (1.27%), respectively. In the control group, 47 cases (7.83%) with the heterozygous p.V37I variation were sequenced, and none had homozygous p.V37I variation or compound p.V37I plus other *GJB2* pathogenic mutation. The distribution of p.V37I homozygous and compound variation in the patient groups was significantly higher than that in the control group (P<0.005), suggesting that the presence of p.V37I homozygous and compound variation was more frequently associated with hearing loss (Table 2).

**Table 1:** Distribution of Compound p.V37I Plus other GJB2 Pathogenic Mutation in the Patient Group.

Allele 1		Allele 2		Cases
Nucleotide Change	Amino acid Change	Nucleotide Change	Amino acid Change	
c.109G>A	p.V37I	c.235delC	Frameshift	32
c.109G>A	p.V37I	c.299delAT	Frameshift	7
c.109G>A	p.V37I	c.427C>T	p.R143W	5
c.109G>A	p.V37I	c.176del16	Frameshift	1
c.109G>A	p.V37I	c.512insAACG	Frameshift	1
c.109G>A	p.V37I	c.257C>G	p.T86R	2
c.109G>A	p.V37I	c.230G>A	p.W77*	1

**Table 2:** Distribution of the p.V37I Variation in the Patient and Control Groups.

Group	p.V37I /-	p.V37I / p.V37I	p.V37I /other Mutation	No p.V37I Variation	Total
Patient	208 (5.38%)	57 (1.48%)	49 (1.27%)	3550 (91.87%)	3864
Control	47 (7.83%)	0 (0%)	0 (0%)	553 (92.17%)	600

The hearing loss phenotype ranged from mild to profound in the cases with homozygous p.V37I variation or compound p.V37I plus other GJB2 pathogenic mutation, and there was no difference in the distribution of the hearing level between the two groups. However, most patients with homozygous p.V37I variation or compound p.V37I plus other *GJB2* pathogenic mutation had a mild or moderate hearing level (66.04%, 70/106) (Table 3).

**Table 3:** Hearing Levels in the Patient Groups with the Homozygous and Compound p.V37I Variant.

Hearing level	p.V37I / p.V37I	p.V37I /other mutation	Total
Mild	9	4	13 (12.26%)
Moderate	32	25	57 (53.78%)
Severe	5	10	15 (14.15%)
Profound	11	10	21 (19.81%)
Total	57	49	106

The Friedman rank-sum test (M-test) was used to identify significant differences in hearing level among the groups, and H = 0.4375, P>0.25.

## Discussion

The c.109G>A variation in *GJB2* is highly prevalent in East Asian deaf patients—11.1% in Thai deafness [7], 16.5% in Japanese deafness [8], 19.4% in Korean deafness [9], and 6.7% in Chinese deafness [10]. The c.109G>A variation, which causes a valine to isoleucine replacement (p.V37I) at position 37, was first reported in 1998 by Kelley [11]. Kelley’s study suggested that p.V37I variation occurred in the M1 domain like p.V27I (c.79G>A, polymorphic change) found only in unaffected controls and speculated that p.V37I might be a polymorphic change. Thus, p.V37I was originally reported as a polymorphism in some studies. However, with the increasing awareness of *GJB2*, reports have increasingly considered p.V37I to be pathogenic with a mild-to-moderate hearing impairment phenotype. Dai’s study[10] revealed that p.V37I had an allele frequency significantly higher than that in the normal population, supported p.V37I as a pathogenic mutation, but homozygous p.V37I variation or compound p.V37I plus other GJB2 pathogenic mutation was rare. Dahl [12] concluded that p.V37I could be a causative mutation associated with slight/mild sensorineural haring loss in Australia. Kim [9] reported that the carrier frequency of p.V37I among Koreans with mild hearing loss (18.2%) were significantly higher than those with severe-profound hearing loss (1.2%) or normal individuals (1.0%), suggesting the contribution of p.V37I to the pathogenesis of milder hearing loss. Chai [13] assessed the prevalence of homozygous p.V37I in large numbers of hearing-impaired and normal subjects, demonstrated that the homozygous p.V37I variant is associated with a broad spectrum of hearing phenotypes, ranging from severe-to-profound hearing loss to normal hearing, and from congenital onset to delayed-onset. Bruzzone [14] found that the valine to isoleucine substitution at position 37(p.V37I) was devoid of functional activity, suggested p.V37I caused hearing loss.

In the present study, 47 (7.83%) patients had all types of p.V37I variation; this frequency was slightly higher than that in Dai’s study (6.7%), likely because most patients in Dai’s study were severe-to-profound hearing impairment. The distribution of homozygous p.V37I variation or compound p.V37I plus other *GJB2* pathogenic mutation (2.75%) in the patient groups was significantly higher than that in the control group (0%) (Table 2), suggesting that the presence of p.V37I variation was more frequently associated with hearing loss. In other studies, a certain proportion of the normal-hearing population has homozygous p.V37I variation or compound p.V37I plus other *GJB2* pathogenic mutation. It was surprising that there was no homozygous p.V37I variation or compound p.V37I plus other *GJB2* pathogenic mutation in our 1,000 control individuals. The reason for the absence of the pathogenic p.V37I combination in the large control group in the present study might be the strict inclusion criteria for the normal controls and their higher average age. Each candidate control individual underwent pure-tone audiometry and would be excluded from the control group if they had even mild hearing loss. The average age of the 600 control individuals was 32 years, and the hearing level of the normal young people who carried the p.V37I mutation might decline with age.

The hearing loss phenotype ranged from mild to profound in the group with bi-allelic *GJB2* mutations containing at least one p.V37I variation. Although there was no difference in the distribution of the hearing level in the patients with homozygous p.V37I variation or compound p.V37I plus other *GJB2* pathogenic mutation, most patients with the p.V37I -homozygous variation or compound p.V37I plus other *GJB2* pathogenic mutation had a mild or moderate hearing level (66.04%, Table 3). Tsukada et al. [9] concluded that, in comparison with the milder p.V37I allele, the hearing level of p.V37I /p.R143W was worse than that of p.V37I / p.V37I, suggesting that p.R143W led to a worse phenotype. In that study, there were five patients with p.V37I /p.R143W mutation—four had severe or profound hearing loss, and one had moderate hearing loss. However, in our study, 28.07% patients with the p.V37I -homozygous variation had severe-profound hearing impairment, and 40.81% with compound p.V37I plus other *GJB2* pathogenic mutation had severe-profound hearing impairment. Although there was no significant difference, the hearing level of most patients with compound p.V37I plus other *GJB2* pathogenic mutation was worse than that of those with the p.V37I -homozygous variation.

**Fig 1:**
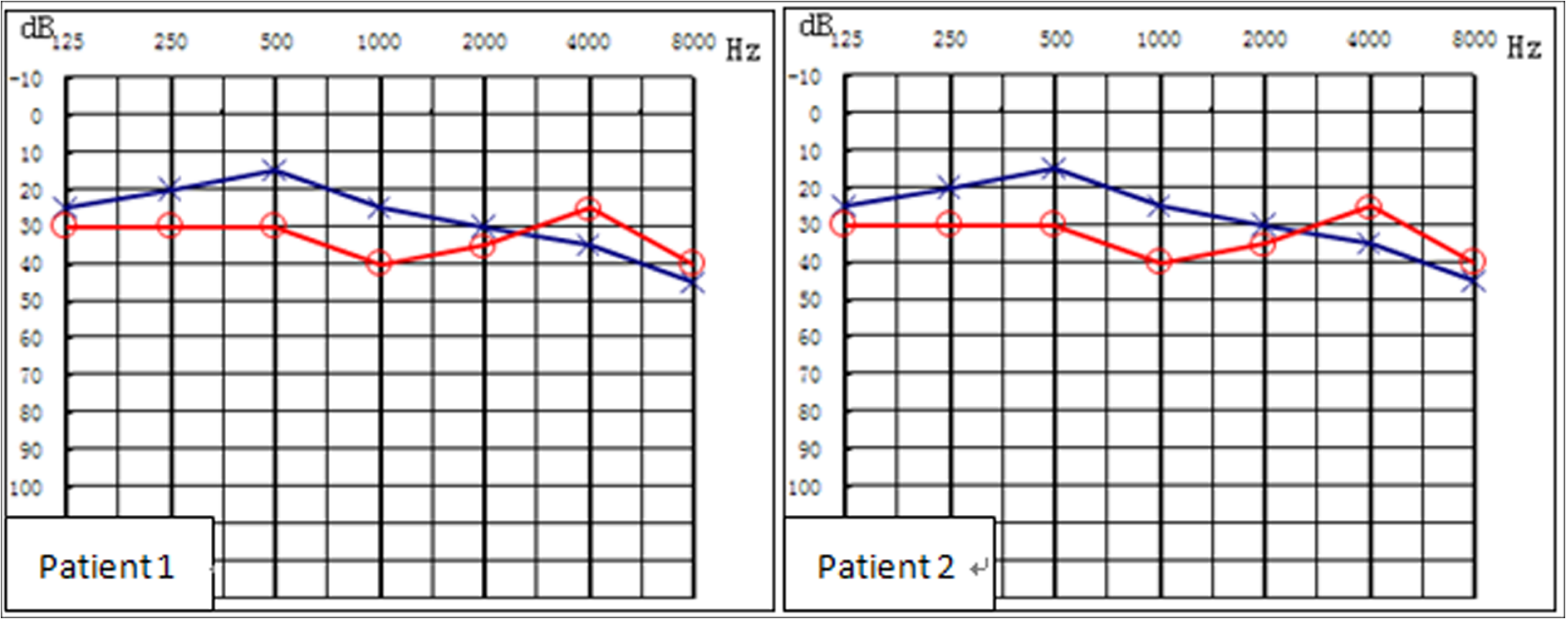
Hearing levels of patients with the p.V37I/c.235delC compound heterozygous variation.

Two studies [12, 14] have assessed homozygous p.V37I in normal subjects. However, among those at our clinics, there was no normal individual with p.V37I homozygous mutation or compound p.V37I plus other GJB2 pathogenic mutation. Some individuals claimed that they had normal hearing with p.V37I /c.235delC compound heterozygous mutation at the clinics, but they were confirmed to have mild hearing impairment through hearing testing (Fig 1). Thus, the results of this study suggested that some patients with p.V37I variation might have had normal hearing by self-assessment, but slight/mild hearing impairment by pure-tone audiometry.

Because the p.V37I variant has been reported to be associated with delayed onset of hearing impairment, Li [15] indicated that the p.V37I exclusive genotype of *GJB2* might cause subclinical hearing impairment at birth and increase the risk for postnatal permanent childhood hearing impairment. In our study, this phenomenon of delayed hearing loss was not evaluated. Thus, we speculated according to our observations that p.V37I caused milder congenital hearing loss in some individuals that might not be noticed until an older age.

However, although the p.V37I mutation was highly prevalent in hearing loss patients, the cause may be the milder phenotype and non-progression of patients with the mutation who either did not visit an otolaryngology clinic or did not receive a recommendation for genetic testing. Therefore, otolaryngologists should consider that the milder phenotype could be caused by the p.V37I mutation.

According to this study and previous reports, there is a broad spectrum of hearing phenotypes caused by the p.V37I variation [9, 12, 13, 15], and it may be influenced by yet-to-be-discovered genetic and environmental factors. In Snoeckx’s multicenter study [16], they performed cross-sectional analyses of *GJB2* genotype and audiometric data from 1,531 patients with mild to profound nonsydromic hearing impairment, found the wide phenotypic variability of DFNB1 deafness, even among subjects homozygous for the same mutation, and it suggested that this variability might reflect the effect of modifier gene and/or environmental factors that lead to incomplete penetrance and variable expression. And this view also was reflected in other literature [17], which pointed out that phenotypes often depended on genetic background, implying that genetic modifiers had a role in guiding the functional consequences of genetic variation. Based on this assumption of the influence of modifier genes, Hilgert’s study [18] performed a whole-genome association study on c.35delG homozygotes in *GJB2*, found some single-nucleotide polymorphisms (SNPs), but did not reveal a major modifier gene, and it suggested that the phenotypic variability in c.35delG homozygous patients could not be explained by the effect of one major modifier gene and the significantly associated SNPs might reflect a small modifying effect on the phenotype. In addition, some papers reported that sensorineural hearing loss could be caused by mutations in two alleles of both different genes. There are reports of patients with sensorineural hearing loss caused by co-existing mutations in GJB2 or SLC26A4 and the mitochondrial gene[19], and one enlarged vestibular aqueduct syndrome patient with compound mutations in both SLC26A4 and GJB2 respectively[20]. So there may be the hypothesis that the hearing impairments of a few cases with homozygous or compound heterozygous p.V37I might have been caused by mutations in other genes simultaneously.

Because of the higher carriage rate of p.V37I in normal individuals, study of its role in pathogenesis will have a marked effect on the diagnosis strategy. To confirm the pathogenicity of p.V37I, comparative studies and epidemiological research with a large sample size are necessary. By evaluating subjects with the p.V37I variation and other mutations, this study found a definite relationship between p.V37I and deafness, and the majority of patients who carried the p.V37I mutation had mild or moderate hearing loss. The results of this study will facilitate analysis of the molecular etiology of patients with deafness of various phenotypes.

## Supporting Information

S1 Text The variants of GJB2 in the study. (DOC)

S2 Text The hearing and mutation in the group of p.V37I-p.V37I. (DOC)

S3 Text The hearing and mutation in the group of p.V37I-other mutation. (DOCX)
