# Supplementary material for: The Relationship between the p.V37I Mutation in GJB2 and Hearing Phenotypes in Chinese Individuals
Source: PLoS One. 2015 Jun 10;10(6):e0129662. doi: 10.1371/journal.pone.0129662 (PMC4463851; doi:10.1371/journal.pone.0129662)
Supplement: S1 Text — (DOC) [file pone.0129662.s001.doc]

| S 1 Text. The mutations and allele frequencies in GJB2 | | |
| --- | --- | --- |
| nucleotide change | amino acid change | Frequencies |
| c.235delC | Frameshift | 90.40% |
| c.299delAT | Frameshift | 28.90% |
| c.176del16 | Frameshift | 7.80% |
| c.257C>G | p.T86R | 5.50% |
| c.512insAACG | Frameshift | 4.70% |
| c.427C>T | p.R143W | 3.60% |
| c.605ins46 | Frameshift | 2.30% |
| c.9G>A | p.W3* | 2.10% |
| c.35insG | Frameshift | 1.80% |
| c.139G>T | p.E47* | 1.00% |
| c.35delG | Frameshift | 0.50% |
| c.95G>A | p.R32H | 0.50% |
| c.ivs1+1G>A | aberrant splicing | 0.50% |
| c.551G>A | p.R184Q | 0.50% |
| c.94C>T | p.R32C | 0.30% |
| c.283G>A | p.V95M | 0.30% |
| c.134G>A | p.G45E | 0.30% |
| c.313del14 | Frameshift | 0.30% |
| c.157T>A | p.C53S | 0.30% |
| c.230G>A | p.W77* | 0.30% |
