# Supplementary material for: The Relationship between the p.V37I Mutation in GJB2 and Hearing Phenotypes in Chinese Individuals
Source: PLoS One. 2015 Jun 10;10(6):e0129662. doi: 10.1371/journal.pone.0129662 (PMC4463851; doi:10.1371/journal.pone.0129662)
Supplement: S2 Text — (DOC) [file pone.0129662.s002.doc]

S 2 Text. The hearing and mutation in the group of p.V37I-p.V37I

|  |  |  |  |  | Left ear | | | | | | |  | Left ear | | | | | | |
| --- | --- | --- | --- | --- | --- | --- | --- | --- | --- | --- | --- | --- | --- | --- | --- | --- | --- | --- | --- |
| samle | sex | age | gene mutation | CT | 0.25L | 0.5L | 1 | 2 | 4 | 8 | PTA |  | 0.25 | 0.5R | 1 | 2 | 4 | 8 | PTA |
| 0566 | W | 44 | p.V37I/p.V37I | N |  | 45 | 50 | 45 | 60 |  | 47.5 |  |  | 50 | 40 | 40 | 65 |  | 48.75 |
| 0570 | M | 32 | p.V37I/p.V37I | N | 90 | 100 | 100 | 100 | 100 | 96 | 100 |  | 90 | 95 | 90 | 85 | 90 | 70 | 90 |
| 0657 | W | 8 | p.V37I/p.V37I | N | 80 | 85 | 105 | 120 | 115 | 100 | 106 |  | 65 | 70 | 80 | 95 | 115 | 105 | 90 |
| 0722 | W | 9 | p.V37I/p.V37I | N | 30 | 25 | 20 | 30 | 50 | 80 | 31.25 |  | 55 | 50 | 45 | 45 | 45 | 70 | 46.25 |
| 0751 | M | 30 | p.V37I/p.V37I | N | 25 | 25 | 30 | 35 | 40 | 70 | 32.5 |  | 20 | 30 | 25 | 45 | 55 | 65 | 38.75 |
| 0860 | M | 23 | p.V37I/p.V37I | N | 40 | 45 | 35 | 30 | 25 | 40 | 33.75 |  | 75 | 70 | 75 | 85 | 80 | 75 | 77.5 |
| 0880 | M | 16 | p.V37I/p.V37I | N | 40 | 50 | 60 | 70 | 85 | 90 | 66.25 |  | 15 | 25 | 35 | 45 | 55 | 40 | 40 |
| 0939 | W | 30 | p.V37I/p.V37I | N | 45 | 50 | 50 | 75 | 90 | 90 | 66.25 |  | 55 | 50 | 55 | 80 | 80 | 65 | 66.25 |
| 1011 | M | 6 | p.V37I/p.V37I | N | 20 | 40 | 45 | 40 | 60 | 80 | 46.25 |  | 90 | 100 | 100 | 95 | 90 | 85 | 96.25 |
| 1049 | W | 35 | p.V37I/p.V37I | N | 30 | 35 | 55 | 55 | 45 | 75 | 47.5 |  | 35 | 40 | 55 | 85 | 65 | 95 | 61.25 |
| 1378 | M | 1.3 | p.V37I/p.V37I | N | 30 | 35 | 35 | 40 | 40 | 35 | 37.5 |  | 95 | 90 | 85 | 75 | 80 | 70 | 82.5 |
| 1395 | W | 3 | p.V37I/p.V37I | N |  | 65 | 70 | 70 | 50 |  | 63.75 |  |  | 95 | 80 | 85 | 80 |  | 85 |
| 1419 | W | 2 | p.V37I/p.V37I | N | 55 | 55 | 70 | 65 | 70 | 55 | 65 |  | 30 | 35 | 75 | 60 | 60 | 60 | 57.5 |
| 1475 | M | 27 | p.V37I/p.V37I | N | 40 | 30 | 20 | 65 | 70 | 70 | 46.25 |  | 45 | 35 | 45 | 55 | 55 | 65 | 47.5 |
| 1513 | M | 0.7 | p.V37I/p.V37I | N |  |  |  |  |  |  | 100 |  |  |  |  |  |  |  | 90 |
| 1549 | M | 67 | p.V37I/p.V37I | N | 30 | 45 | 60 | 55 | 70 | 100 | 57.5 |  | 35 | 45 | 50 | 65 | 80 | 100 | 60 |
| 1600 | M | 0.7 | p.V37I/p.V37I | N |  | 40 | 40 | 40 | 60 |  | 45 |  |  | 50 | 25 | 50 | 60 |  | 46.25 |
| 1961 | W | 5 | p.V37I/p.V37I | N | 90 | 100 | 105 | 110 | 115 |  | 107.5 |  | 85 | 85 | 75 | 75 | 65 |  | 75 |
| 2138 | W | 46 | p.V37I/p.V37I | N | 20 | 20 | 30 | 60 | 60 | 70 | 42.5 |  | 30 | 30 | 35 | 45 | 50 | 75 | 40 |
| 2179 | M | 22 | p.V37I/p.V37I | N | 25 | 25 | 45 | 70 | 70 | 60 | 52.5 |  | 20 | 30 | 50 | 70 | 70 | 60 | 55 |
| 2248 | W | 32 | p.V37I/p.V37I | N | 20 | 35 | 40 | 40 | 55 | 65 | 42.5 |  | 30 | 30 | 45 | 40 | 45 | 55 | 40 |
| 2295 | M | 1 | p.V37I/p.V37I | N |  | 95 | 100 | 100 | 100 |  | 98.75 |  |  | 75 | 90 | 95 | 90 |  | 87.5 |
| 2349 | M | 21 | p.V37I/p.V37I | N | 45 | 55 | 55 | 60 | 50 | 60 | 55 |  | 45 | 50 | 70 | 70 | 60 | 65 | 62.5 |
| 2395 | M | 8 | p.V37I/p.V37I | N | 25 | 30 | 35 | 55 | 55 | 50 | 43.75 |  | 40 | 40 | 50 | 45 | 55 | 55 | 47.5 |
| 2473 | M | 20 | p.V37I/p.V37I | N | 15 | 15 | 35 | 45 | 40 | 45 | 33.75 |  | 15 | 15 | 25 | 45 | 50 | 50 | 33.75 |
| 2555 | W | 1 | p.V37I/p.V37I | N |  | 15 | 20 | 45 | 50 |  | 32.5 |  |  | 25 | 20 | 35 | 50 |  | 32.5 |
| 2627 | W | 7 | p.V37I/p.V37I | N | 55 | 60 | 60 | 55 | 60 | 50 | 58.75 |  | 50 | 55 | 50 | 55 | 80 | 65 | 60 |
| 2650 | M | 1 | p.V37I/p.V37I | N |  | 95 | 110 | 110 | 110 |  | 106.3 |  |  | 65 | 90 | 90 | 70 |  | 78.75 |
| 2843 | W | 17 | p.V37I/p.V37I | N | 40 | 30 | 60 | 85 | 80 | 90 | 63.75 |  | 30 | 25 | 50 | 85 | 80 | 75 | 60 |
| 2874 | W | 31 | p.V37I/p.V37I | N | 60 | 60 | 65 | 60 | 50 | 50 | 58.75 |  | 20 | 45 | 55 | 65 | 45 | 40 | 52.5 |
| 2944 | M | 34 | p.V37I/p.V37I | N | 15 | 25 | 30 | 50 | 65 | 65 | 42.5 |  | 15 | 20 | 30 | 45 | 50 | 70 | 36.25 |
| 3393 | W | 16 | p.V37I/p.V37I | N | 50 | 50 | 50 | 50 | 50 | 45 | 50 |  | 50 | 50 | 50 | 45 | 50 | 35 | 48.75 |
| 3774 | M | 1 | p.V37I/p.V37I | N |  |  |  |  |  |  | 55 |  |  |  |  |  |  |  | 55 |
| 3839 | M | 24 | p.V37I/p.V37I | N | 45 | 45 | 45 | 55 | 70 | 90 | 53.75 |  | 45 | 50 | 45 | 50 | 65 | 85 | 52.5 |
| 4048 | W | 10 | p.V37I/p.V37I | N | 15 | 25 | 30 | 40 | 45 | 50 | 35 |  | 10 | 20 | 20 | 55 | 45 | 45 | 35 |
| 4079 | M | 22 | p.V37I/p.V37I | N | 85 | 100 | 110 | 120 | 120 | 105 | 112.5 |  | 95 | 90 | 105 | 120 | 110 | 105 | 106.25 |
| 4307 | M | 4 | p.V37I/p.V37I | N |  |  |  |  |  |  | 95 |  |  |  |  |  |  |  | 100 |
| 4328 | M | 23 | p.V37I/p.V37I | N | 20 | 40 | 50 | 45 | 50 | 55 | 46.25 |  | 20 | 60 | 70 | 65 | 60 | 60 | 63.75 |
| 4390 | M | 6 | p.V37I/p.V37I | N | 45 | 50 | 45 | 40 | 45 | 80 | 45 |  | 35 | 35 | 35 | 40 | 35 | 90 | 36.25 |
| 4507 | M | 10 | p.V37I/p.V37I | N | 20 | 25 | 25 | 50 | 50 | 55 | 37.5 |  | 20 | 25 | 30 | 50 | 50 | 70 | 38.75 |
| 4578 | M | 9M | p.V37I/p.V37I | N |  | 70 | 85 | 90 | 90 |  | 83.75 |  |  | 45 | 60 | 65 | 60 |  | 57.5 |
| 4657 | W | 44 | p.V37I/p.V37I | N | 25 | 30 | 40 | 45 | 55 | 75 | 42.5 |  | 25 | 35 | 45 | 45 | 55 | 75 | 45 |
| 4724 | M | 6 | p.V37I/p.V37I | N |  | 110 | 105 | 110 | 110 |  | 108.8 |  |  | 110 | 105 | 110 | 110 |  | 108.75 |
| 4773 | W | 9 | p.V37I/p.V37I | N | 80 | 85 | 80 | 75 | 70 | 80 | 77.5 |  | 65 | 65 | 60 | 55 | 50 | 55 | 57.5 |
| 5216 | M | 33 | p.V37I/p.V37I | N | 65 | 60 | 70 | 90 | 80 | 70 | 75 |  | 70 | 65 | 60 | 60 | 60 | 65 | 61.25 |
| 5378 | M | 4 | p.V37I/p.V37I | N |  |  |  |  |  |  | 80 |  |  |  |  |  |  |  | 60 |
| 5449 | M | 6 | p.V37I/p.V37I | N |  | 75 | 80 | 65 | 60 |  | 70 |  |  | 55 | 80 | 80 | 50 |  | 66.25 |
| 5457 | W | 16 | p.V37I/p.V37I | N | 35 | 45 | 40 | 55 | 50 | 55 | 47.5 |  | 45 | 50 | 50 | 50 | 50 | 45 | 50 |
| 5473 | M | 8M | p.V37I/p.V37I | N |  | 35 | 30 | 40 | 50 |  | 38.75 |  |  | 35 | 30 | 40 | 50 |  | 38.75 |
| 5506 | W | 39 | p.V37I/p.V37I | N | 25 | 35 | 40 | 70 | 65 | 70 | 52.5 |  | 30 | 40 | 50 | 70 | 70 | 65 | 57.5 |
| 5566 | M | 3 | p.V37I/p.V37I | N | 75 | 75 | 75 | 60 | 65 | 95 | 68.75 |  | 95 | 95 | 95 | 85 | 85 | 100 | 90 |
| 5589 | M | 20 | p.V37I/p.V37I | N | 35 | 35 | 35 | 30 | 45 | 40 | 36.25 |  | 50 | 45 | 50 | 50 | 55 | 50 | 50 |
| 6090 | W | 4 | p.V37I/p.V37I | N | 80 | 100 | 100 | 100 | 100 | 100 | 100 |  | 100 | 100 | 100 | 100 | 100 | 100 | 100 |
| 6236 | M | 8 | p.V37I/p.V37I | N |  | 100 | 100 | 100 | 100 |  | 100 |  |  | 100 | 100 | 100 | 100 |  | 100 |
| 6550 | M | 4 | p.V37I/p.V37I | N | 90 | 90 | 80 | 70 | 100 |  | 85 |  | 100 | 100 | 100 | 100 | 70 |  | 92.5 |
| 301333 | W | 4 | p.V37I/p.V37I | N | 25 | 30 | 40 | 60 | 50 |  | 45 |  | 25 | 25 | 35 | 55 | 55 |  | 42.5 |
| 301714 | W | 1 | p.V37I/p.V37I | N | 15 | 15 | 20 | 40 | 35 | 30 | 27.5 |  | 10 | 20 | 35 | 35 | 35 | 30 | 31.25 |
