# Supplementary material for: The Relationship between the p.V37I Mutation in GJB2 and Hearing Phenotypes in Chinese Individuals
Source: PLoS One. 2015 Jun 10;10(6):e0129662. doi: 10.1371/journal.pone.0129662 (PMC4463851; doi:10.1371/journal.pone.0129662)
Supplement: S3 Text — (DOCX) [file pone.0129662.s003.docx]

S 3 Table. The hearing and mutation in the group of p.V37I-other mutation

|  |  |  |  |  | Left ear | | | | | | |  | right ear | | | | | | |
| --- | --- | --- | --- | --- | --- | --- | --- | --- | --- | --- | --- | --- | --- | --- | --- | --- | --- | --- | --- |
| sample | sex | age | gene mutation | CT | 250 | 500 | 1000 | 2000 | 4000 | 8000 | PTA |  | 250 | 500 | 1000 | 2000 | 4000 | 8000 | PTA |
| 0490 | M | 26 | p.V37I/c.235delC | N | 30 | 40 | 45 | 50 | 50 | 45 | 46.25 |  | 30 | 40 | 55 | 75 | 60 | 75 | 57.5 |
| 0703 | M | 14 | p.V37I/c.235delC | N | 30 | 40 | 50 | 45 | 35 | 55 | 42.5 |  | 55 | 60 | 50 | 55 | 45 | 50 | 52.5 |
| 0705 | W | 19 | p.V37I/p.W77* | N | 10 | 20 | 30 | 40 | 50 | 70 | 35 |  | 15 | 25 | 35 | 50 | 35 | 70 | 36.25 |
| 0775 | M | 51 | p.V37I/c.176del16 | N | 65 | 70 | 60 | 75 | 90 | 90 | 73.75 |  | 50 | 55 | 65 | 65 | 70 | 90 | 63.75 |
| 0901 | M | 7 | p.V37I/c.235delC | N |  |  |  |  |  |  | 80 |  |  |  |  |  |  |  | 50 |
| 0905 | M | 2 | p.V37I/c.235delC | N | 85 | 85 | 90 | 80 | 105 | 105 | 90 |  | 80 | 85 | 90 | 80 | 85 | 105 | 85 |
| 1003 | M | 3 | p.V37I/c.235delC | N | 90 | 100 | 105 | 105 | 110 | 105 | 105 |  | 95 | 95 | 110 | 110 | 110 | 105 | 106.3 |
| 1254 | M | 7 | p.V37I/c.299delAT | N | 45 | 50 | 50 | 75 | 55 | 65 | 57.5 |  | 40 | 40 | 50 | 55 | 60 | 50 | 51.25 |
| 1615 | M | 30 | p.V37I/c.235delC | N | 20 | 20 | 20 | 25 | 25 | 45 | 22.5 |  | 45 | 45 | 40 | 50 | 40 | 50 | 43.75 |
| 1743 | W | 2 | p.V37I/c.235delC | N | 70 | 80 | 95 | 90 | 95 | 90 | 90 |  | 60 | 80 | 110 | 100 | 105 | 100 | 98.75 |
| 1943 | M | 11 | p.V37I/c.235delC | N | 75 | 75 | 90 | 95 | 90 | 105 | 87.5 |  | 35 | 40 | 50 | 60 | 80 | 105 | 57.5 |
| 2164 | M | 20 | p.V37I/c.235delC | N | 85 | 85 | 90 | 85 | 70 | 90 | 82.5 |  | 70 | 80 | 85 | 80 | 75 | 90 | 80 |
| 2259 | W | 7 | p.V37I/c.235delC | N | 60 | 65 | 65 | 60 | 40 | 50 | 57.5 |  | 60 | 65 | 65 | 55 | 55 | 55 | 60 |
| 2296 | W | 33 | p.V37I/c.235delC | N | 50 | 55 | 65 | 70 | 70 | 105 | 65 |  | 80 | 80 | 80 | 75 | 70 | 70 | 76.25 |
| 2410 | M | 16 | p.V37I/p.R143W | N | 50 | 60 | 70 | 60 | 65 | 65 | 63.75 |  | 65 | 60 | 65 | 60 | 65 | 65 | 62.5 |
| 2676 | W | 44 | p.V37I/c.235delC | N | 55 | 50 | 55 | 70 | 90 | 105 | 66.25 |  | 40 | 40 | 35 | 45 | 50 | 70 | 42.5 |
| 2700 | M | 14 | p.V37I/p.R143W | N | 50 | 55 | 50 | 55 | 45 | 60 | 51.25 |  | 50 | 50 | 50 | 50 | 45 | 50 | 48.75 |
| 2792 | W | 1 | p.V37I/c.235delC | N |  |  |  |  |  |  | 70 |  |  |  |  |  |  |  | 70 |
| 2832 | W | 9 | p.V37I/c.235delC | N | 60 | 50 | 55 | 50 | 40 | 55 | 48.75 |  | 60 | 60 | 90 | 85 | 95 | 70 | 82.5 |
| 2865 | W | 51 | p.V37I/c.235delC | N | 45 | 40 | 60 | 65 | 60 | 60 | 56.25 |  | 35 | 55 | 60 | 70 | 65 | 60 | 62.5 |
| 3030 | W | 6 | p.V37I/c.235delC | N | 40 | 45 | 65 | 65 | 55 | 40 | 57.5 |  | 55 | 45 | 50 | 50 | 40 | 40 | 46.25 |
| 3101 | W | 38 | p.V37I/c.235delC | N | 25 | 20 | 30 | 55 | 75 | 75 | 45 |  | 20 | 15 | 25 | 40 | 45 | 45 | 31.25 |
| 3403 | M | 9 | p.V37I/c.299delAT | N | 30 | 35 | 35 | 40 | 50 | 55 | 40 |  | 40 | 45 | 50 | 55 | 55 | 50 | 51.25 |
| 3437 | W | 2 | p.V37I/c.235delC | N | 45 | 50 | 60 | 65 | 90 | 70 | 66.25 |  | 85 | 75 | 80 | 75 | 80 | 90 | 77.5 |
| 3455 | W | 11 | p.V37I/c.235delC | N | 20 | 20 | 10 | 20 | 65 | 60 | 28.75 |  | 105 | 110 | 95 | 110 | 105 | 105 | 105 |
| 3506 | M | 9 | V37I/299delAT | N | 15 | 25 | 45 | 40 | 55 | 50 | 41.25 |  | 25 | 40 | 65 | 65 | 65 | 60 | 58.75 |
| 3607 | M | 4M | p.V37I/c.235delC | N |  | 100 | 90 | 100 | 100 |  | 97.5 |  |  | 90 | 100 | 100 | 100 |  | 97.5 |
| 3632 | M | 18 | p.V37I/c.235delC | N | 50 | 55 | 70 | 65 | 65 | 60 | 63.75 |  | 55 | 60 | 70 | 80 | 75 | 60 | 71.25 |
| 3804 | W | 3 | p.V37I/c.235delC | N | 100 | 110 | 90 | 100 | 90 |  | 97.5 |  | 90 | 90 | 90 | 80 | 70 |  | 82.5 |
| 4170 | M | 5 | p.V37I/p.R143W | N |  | 90 | 100 | 95 | 95 |  | 95 |  |  | 65 | 90 | 100 | 100 |  | 88.75 |
| 4175 | W | 5 | p.V37I/c.235delC | N | 55 | 65 | 60 | 45 | 55 | 70 | 56.25 |  | 55 | 55 | 70 | 60 | 60 | 75 | 61.25 |
| 4210 | W | 4 | p.V37I/p.T86R | N |  |  |  |  |  |  | 120 |  |  |  |  |  |  |  | 105 |
| 4282 | M | 8M | V37I/299delAT | N |  |  |  |  |  |  | 100 |  |  |  |  |  |  |  | 100 |
| 4363 | M | 4 | p.V37I/c.235delC | N | 60 | 50 | 35 | 45 | 70 | 60 | 50 |  | 45 | 40 | 45 | 50 | 65 | 70 | 50 |
| 4373 | W | 37 | p.V37I/c.235delC | N | 40 | 40 | 50 | 40 | 60 | 60 | 47.5 |  | 50 | 50 | 40 | 40 | 50 | 45 | 45 |
| 4573 | W | 3 | p.V37I/c.235delC | N |  |  |  |  |  |  | 103 |  |  |  |  |  |  |  | 103 |
| 4707 | W | 29 | p.V37I/p.T86R | N | 85 | 90 | 90 | 80 | 75 | 60 | 83.75 |  | 70 | 65 | 70 | 60 | 50 | 50 | 61.25 |
| 4953 | M | 21 | p.V37I/p.R143W | N | 55 | 60 | 75 | 80 | 80 | 85 | 73.75 |  | 70 | 70 | 75 | 80 | 80 | 85 | 76.25 |
| 5344 | W | 5 | p.V37I/c.512insAACG | N | 40 | 25 | 35 | 40 | 50 | 35 | 37.5 |  | 45 | 40 | 30 | 45 | 45 | 20 | 40 |
| 5383 | W | 7 | p.V37I/c.235delC | N | 25 | 35 | 40 | 50 | 55 | 50 | 45 |  | 25 | 35 | 40 | 50 | 55 | 50 | 45 |
| 5397 | M | 15 | p.V37I/c.235delC | N | 55 | 60 | 70 | 75 | 60 | 35 | 66.25 |  | 75 | 60 | 70 | 75 | 65 | 55 | 67.5 |
| 5520 | M | 40 | p.V37I/c.299delAT | N | 50 | 50 | 70 | 70 | 60 | 60 | 62.5 |  | 75 | 65 | 60 | 60 | 55 | 60 | 60 |
| 5534 | M | 15 | p.V37I/c.235delC | N | 40 | 40 | 45 | 55 | 40 | 60 | 45 |  | 35 | 35 | 60 | 50 | 35 | 40 | 45 |
| 5813 | M | 28 | p.V37I/c.235delC | N | 50 | 60 | 75 | 85 | 70 | 70 | 72.5 |  | 55 | 65 | 80 | 85 | 70 | 80 | 75 |
| 5828 | M | 12 | p.V37I/c.299delAT | N |  | 95 | 110 | 100 | 100 |  | 101.3 |  |  | 85 | 100 | 100 | 105 |  | 97.5 |
| 5875 | M | 24 | p.V37I/c.299delAT | N | 95 | 90 | 95 | 100 | 100 | 100 | 96.25 |  | 85 | 80 | 100 | 100 | 100 | 105 | 95 |
| 6406 | M | 2 | p.V37I/p.R143W | N |  | 95 | 100 | 100 | 100 |  | 98.75 |  |  | 85 | 90 | 90 | 90 |  | 88.75 |
| 6551 | M | 26 | p.V37I/c.235delC | N | 70 | 90 | 100 | 100 | 100 | 100 | 97.5 |  | 80 | 80 | 85 | 80 | 65 | 85 | 77.5 |
| 301141 | M | 4 | p.V37I/c.235delC | N | 10 | 15 | 10 | 30 | 15 |  | 17.5 |  | 20 | 35 | 25 | 50 | 15 |  | 31.25 |
